# Long-term effects of the COVID-19 pandemic on five mental and psychological disorders: in terms of the number of disease visits, drug consumption, and scale scores

**DOI:** 10.1186/s12888-023-05166-0

**Published:** 2023-09-18

**Authors:** Han-Yu Zhu, Yi-Mo Guo, Zhi-Ming Pan, Yan Wang, Meng-Li Zhang, Ru-Huang Zhu, Zhang-Ping Li, Zhen Wang

**Affiliations:** 1 Department of Neurology, The Quzhou Affiliated Hospital of Wenzhou Medical University, Quzhou People’s Hospital, Quzhou, Zhejiang 324000 China; 2 The Second Clinical College, Wenzhou Medical University, Wenzhou, Zhejiang 325000 China; 3 Department of Neurology, the First Affiliated Hospital of Wenzhou Medical University, Wenzhou, Zhejiang 325000 China; 4 The First Clinical College, Wenzhou Medical University, Wenzhou, Zhejiang 325000 China; 5 Department of Gynecology, the First Affiliated Hospital of Wenzhou Medical University, Wenzhou, Zhejiang 325000 China; 6 The Quzhou Affiliated Hospital of Wenzhou Medical University, Quzhou People’s Hospital, Quzhou, Zhejiang 324000 China

**Keywords:** Mental and psychological diseases, The COVID-19 pandemic, Long-term effects, Epidemiology

## Abstract

**Background:**

COVID-19 caused mild to severe infections in humans. The long-term epidemic environment harms people’s mental health. To explore the impact of the epidemic on people’s mental and psychological conditions, we surveyed in Wenzhou.

**Methods:**

We collected the data of people who visited the First Affiliated Hospital of Wenzhou Medical University for five types of mental and psychological diseases from January 2018 to December 2021. Then, taking December 2019 as the cut-off point, the 48-month data were divided into the pre-epidemic group and the dur-epidemic group. Based on the above data, statistical analysis was done.

**Results:**

From 2018 to 2021, the number of initial diagnoses, the number of disease visits, and drug consumption for these five types of mental and psychological diseases were all on the rise. Compared with the number of disease visits for all disorders in both psychiatry and neurology departments, it was found that the growth rate of these five diseases was higher than the growth rate of all disorders. We found that the number of disease visits, drug consumption, and scale scores after the COVID-19 outbreak were significantly different from those before the outbreak (P < 0.05). And the number of disease visits positively correlated with drug consumption (P < 0.0001, r = 0.9503), which verified the stability of the data.

**Conclusion:**

The epidemic environment has had a long-term and negative impact on people’s mental and psychological conditions. Therefore, whether or not the epidemic is receding, we still need to be concerned about the impact of COVID-19 on mental and psychological health.

**Supplementary Information:**

The online version contains supplementary material available at 10.1186/s12888-023-05166-0.

## Introduction

Between 1896, when Kraepelin published his first formulation of dementia praecox (DP), psychiatric disorders began to gain widespread attention in Europe. 1] Over the past few decades, there has been a growing recognition that psychiatric disorders include both organic psychiatric disorders caused by acute viral encephalitis and lentiviral infection of the central nervous system (CNS) and functional psychiatric disorders such as psychosis, depression, and bipolar disorder (BD); [2] some functional psychiatric disorders are characterized by debilitating symptoms and high recurrence rates, which impose a huge burden on families and society [3–5].

The 2020 COVID-19 pandemic, caused by severe acute respiratory syndrome coronavirus 2 (SARS-CoV-2), was posing an unprecedented crisis worldwide [6]. It has high infectious potential and its incidence rate increases exponentially. Its widespread was regarded as an epidemic by the World Health Organization (WHO). By Dec 31, 2021, global reported deaths due to COVID-19 reached 5.94 million, and the global all-age rate of excess mortality due to the pandemic was 120.3 deaths per 100 000 of the population [7]. In addition to COVID-19-related deaths, the pandemic has also had indirect effects on other health conditions [8].

The COVID-19 pandemic has harmed people’s mental health. Anxiety or depression may be induced due to conditions specific to COVID-19 pandemic periods, such as fear of having SARS-CoV-2, restrictions on going out, substantial financial loss, reduced opportunities to communicate with friends and conflicting information from authorities [9, 10]. Dr. Slavich claimed that major life events in the areas of health, social, work, or finances can cause about half of people to experience depression. And for some, the COVID-19 pandemic destroyed above four domains at once [11]. And studies have shown that a combination of systemic infection, viral neurotropism, and environmental stress promotes or even induces the development of psychiatric disorders, thereby exacerbating the course of the COVID-19 pandemic [2].

From the above, it’s clear that the COVID-19 pandemic has had a dramatic impact on people’s mental health. And so far, research on COVID-19 and mental health has emerged, but the results from previous studies have been inconsistent [12, 13]. Therefore, this study investigated the impact of the COVID-19 pandemic on mental and psychological health from the aspects of disease visits, drug consumption, and scale scores.

## Methods

### Data collection

The basic information of outpatients in the First Affiliated Hospital of Wenzhou Medical University from 2018 to 2021 was collected. The number of inital diagnoses (only the first diagnosis of each patient with the same disease is taken), the number of disease visits (all visits over some time for the same disease were taken), drug consumption, and scale scores of patients with five types of mental and psychological diseases, which including anxiety, depression, sleep disorders, stress disorders, and eating and excretion disorders, were included. Since most of these five types of mental and psychological diseases were chronic diseases, there is a phenomenon that the number of visits increases year by year with the increase of time. Therefore, we also calculated the annual growth rate of initial diagnosis and disease visits. Commonly used anxiolytics, antidepressants, and sleeping pills were selected, such as selective serotonin reuptake inhibitors (SSRI), serotonin-norepinephrine reuptake inhibitor (SNRI), suppressor of activator protein 1, regulated by IFN (SARI), norepinephrine and dopamine reuptake inhibitors (NDRI), tandospirone, Deanxit (DEA), mirtazapine, benzodiazepines, and non-benzodiazepines. The Hamilton Anxiety Scale (HAMA), the Hamilton Depression Scale (HAMD), and the Pittsburgh Sleep Quality Index Scale (PSQI) scores were selected. Then, we chose stratified sampling. We divided the 4 years into 48 months, and each month selected 4 cases from each of the three scales, and then took the average value for statistical analysis. Finally, we took December 2019 as the cut-off point 14] and divided the 48-month disease visits, drug doses, and scale scores into the pre-epidemic group and the dur-epidemic group.

### Statistical analysis

SPSS 26.0 was used for statistical analysis, and GraphPad Prism 8.0.1 was used for data visualization.

First, we statistically described the annual number of inital diagnoses of five types of mental and psychological diseases and scale scores. Based on the background that the number of disease visits is increasing year by year, we compared the growth rate of disease visits for these five types of mental and psychological diseases with the growth rate of disease visits for all types of diseases in psychiatry outpatient clinics and neurology outpatient clinics, to observe the growth of these five types of diseases themselves.

Next, the independent samples T-test was used for normally distributed data and the Mann-Whitney U test for skewed data. Significant analysis was conducted on the number of inital diagnoses of disease, the number of disease visits, the dosage of psychotropic drugs, and the scale scores of the two groups which before or after the outbreak of COVID-19.

In addition, Pearson correlation analysis was used for normally distributed data, Spearman correlation analysis was used for skewed distribution data, and linear regression was used to analyze the correlation between drug consumption and the number of disease visits, which verified the stability of the data.

Finally, the X 2] test was used for categorical data. Five types of mental and psychological diseases patients were stratified by gender and age to explore whether gender or age would affect the existing results.

## Results

**Fig. 1:**
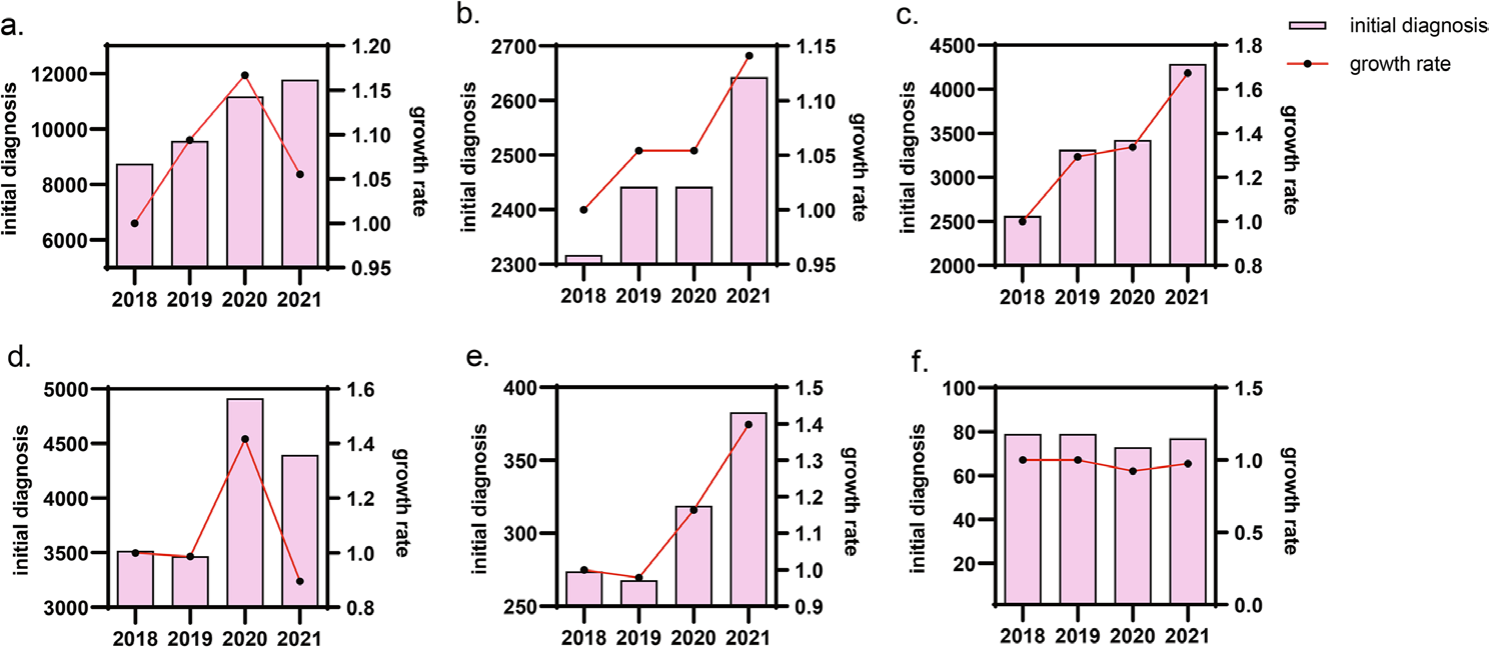
Trends in inital diagnosis of diseases between 2018–2021 Note: Fig. 1**a**. showed the initial diagnosis and growth rate of five types of mental and psychological disorders. Figure 1**b**. and Fig. 1**c**. showed that the annual number of inital diagnoses and growth rate for anxiety and depression disorders were on the rise and both peaked in 2021. Figure 1**d**. showed that inital diagnoses and growth rate for sleep disorders were highest in 2020. Figure 1**e**. showed that both the inital diagnoses and the growth rate of stress disorders were the lowest in 2019, and both showed an upward trend after the epidemic. But from Fig. 1**f**., it can be seen that the annual inital diagnosis and growth rate of eating and excretion disorders within 4 years did not change significantly

Figure 1 and Supplementary Table 1 described the trends of the inital diagnoses (a total of 41,285 person-visits) for the five types of mental and psychological diseases during 2018–2021. Figure 1a. showed that the number of inital diagnoses for the five types of mental and psychological diseases has generally increased year by year in the past 4 years. We found that the annual growth rate of these five types of mental and psychological diseases had a peak in 2020, indicating that excluding the time factor, the inital diagnosis of these diseases was still on the rise in 2020. Figure 1b f. showed the number of initial diagnosis and growth rate of anxiety, depression, sleep disorders, stress disorders, and eating and excretion disorders, respectively.

**Fig. 2:**
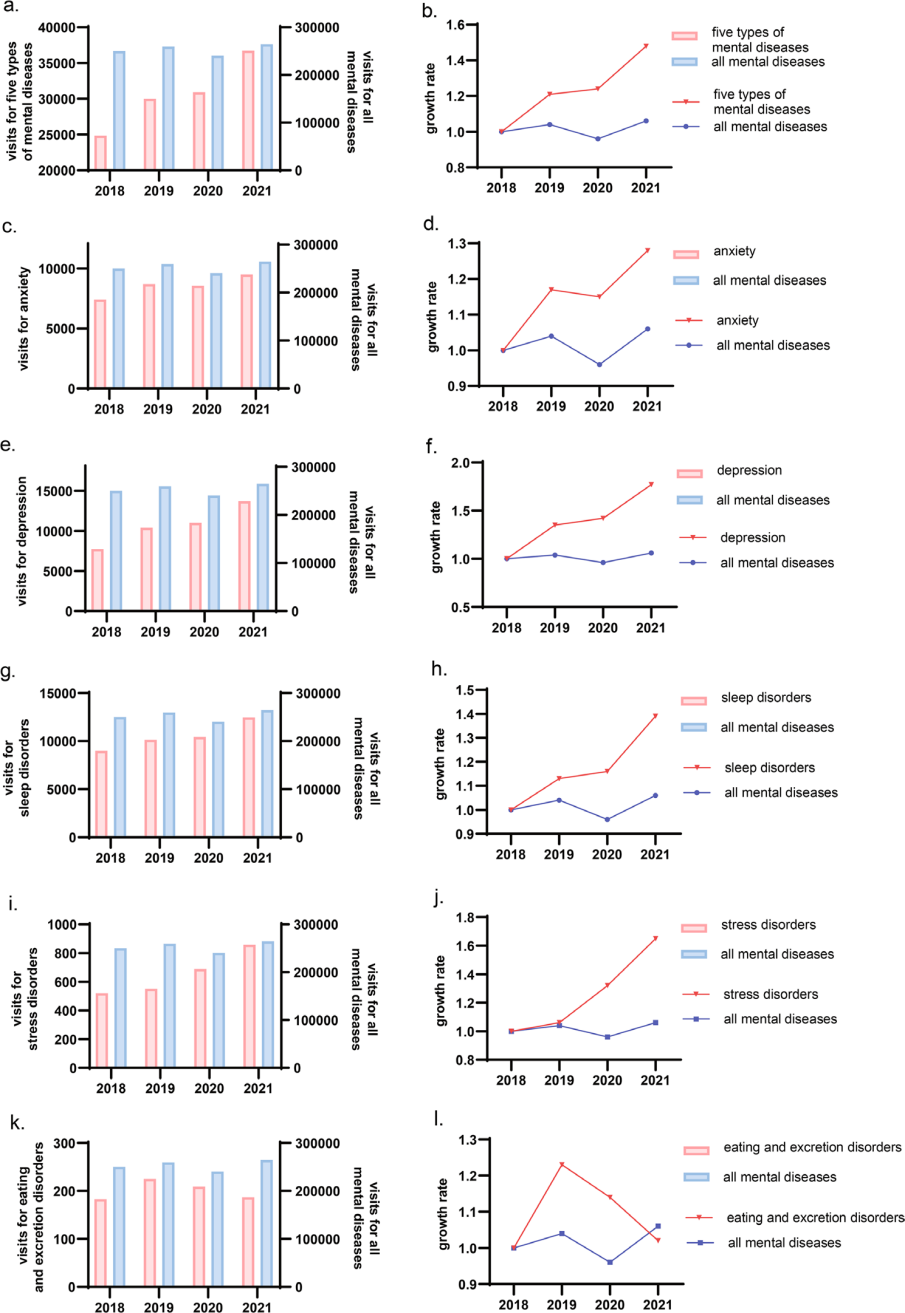
Trends in these five diseases and all mental and psychological diseases visits between 2018–2021 Note: Fig. 2**a**. and Fig. 2**b**. showed the disease visits and growth rate of five types of mental and psychological disorders. Figure 2**c**. and Fig. 2**d**. showed that the number of anxiety visits had a downward trend in 2020, which may be related to the quarantine policies in the early stages of the epidemic. Figure 2**e** and **j**. showed that the number of visits for the three types of mental and psychological diseases, including depression, sleep disorders, and stress disorders, have all been on the rise in 4 years. Figure 2**k**. and Fig. 2**l**. showed that the changing trend of the number of visits for eating and excretion disorders in 4 years was like a peak, and the number of visits showed a downward trend in 2020 and 2021

To compare the performance of these five diseases with all mental and psychological diseases, we did the following analysis. Figure 2 and Supplementary Table 1 described the changing trends of these five diseases and all mental and psychological diseases from 2018 to 2021 (a total of 1,014,929 visits). We found that the number of general department consultations within 4 years had a downward trend in 2020. However, for these five types of mental disorders, the number of disease consultations as well as growth rate of disease visits in the 4 years were on the rise (Fig. 2a. and Fig. 2b.). Figure 2c L. showed the number of disease visits and corresponding growth rate of anxiety, depression, sleep disorders, stress disorders, and eating and excretion disorders, respectively.

**Fig. 3:**
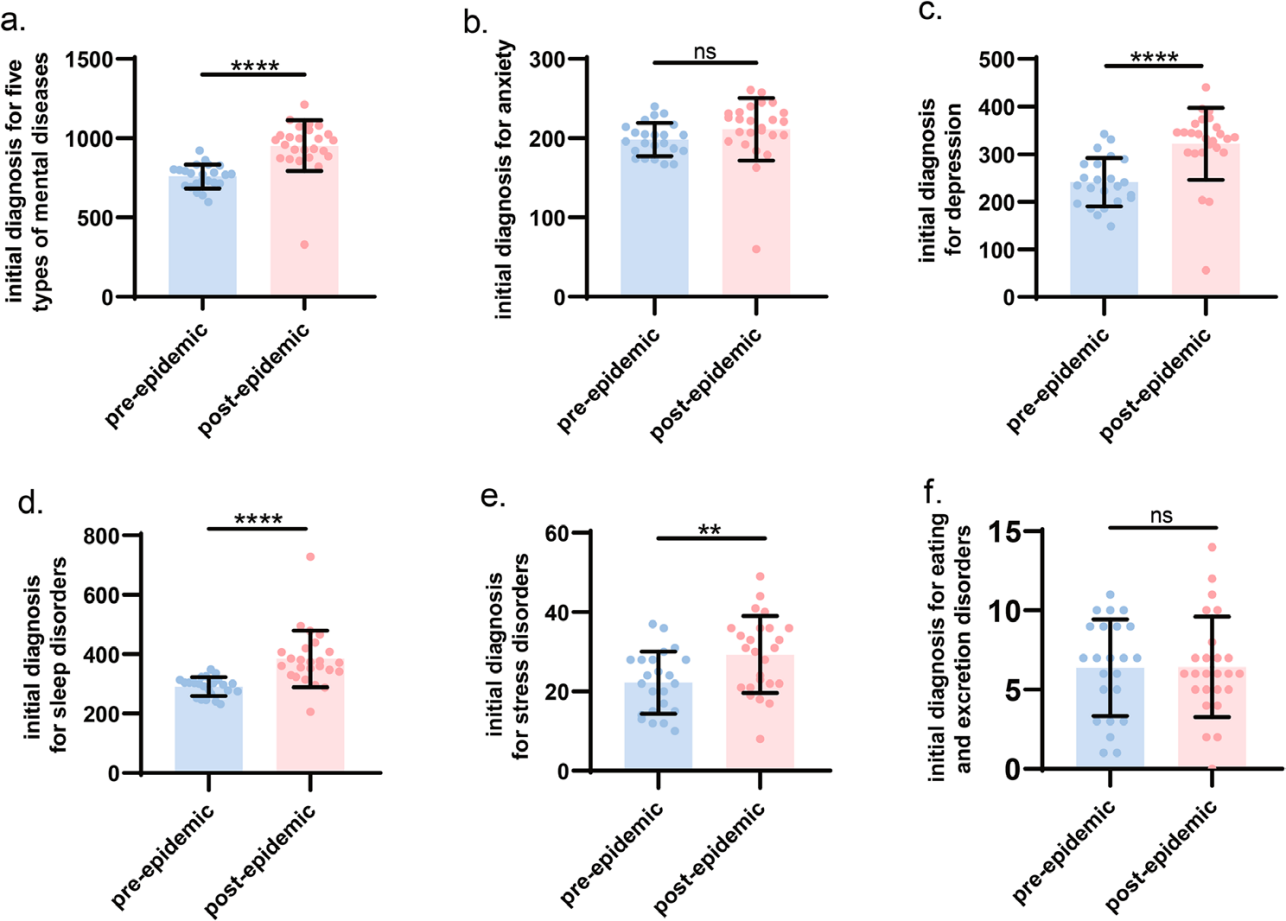
Comparison of the inital diagnosis of diseases in the pre-epidemic group and the dur-epidemic group Note: Fig. 3**a**. showed that there was a significant difference in the number of inital diagnoses for five types of mental and psychological diseases between the pre-epidemic group and the dur-epidemic group (P < 0.0001). Figure 3**c**. Fig. 3**e**. showed that the number of inital diagnoses for the three diseases of depression, sleep disorder, and stress disorders were significantly different between the two groups (P < 0.0078), and the number of inital diagnoses in the dur-epidemic group was higher than that in the pre-epidemic group. Figure 3**b**. and Fig. 3**f**. showed that there was no significant difference in the number of inital diagnoses for anxiety and eating and excretion disorders between these two groups (P > 0.05)

To clarify whether the epidemic will affect the number of inital diagnoses for these five types of mental and psychological diseases, we took December 2019 as the cut-off point and divided them into two groups: the pre-epidemic group and the dur-epidemic group. Figure 3a. showed that there was a significant difference in the number of inital diagnoses for five types of mental and psychological diseases between these two groups (P < 0.0001), and the number of inital diagnoses in the dur-epidemic group was higher than that in the pre-epidemic group. Figure 3b f. showed the differences in the number of initial diagnoses between the two groups for anxiety, depression, sleep disorders, stress disorders, and eating and excretion disorders, respectively.

**Fig. 4:**
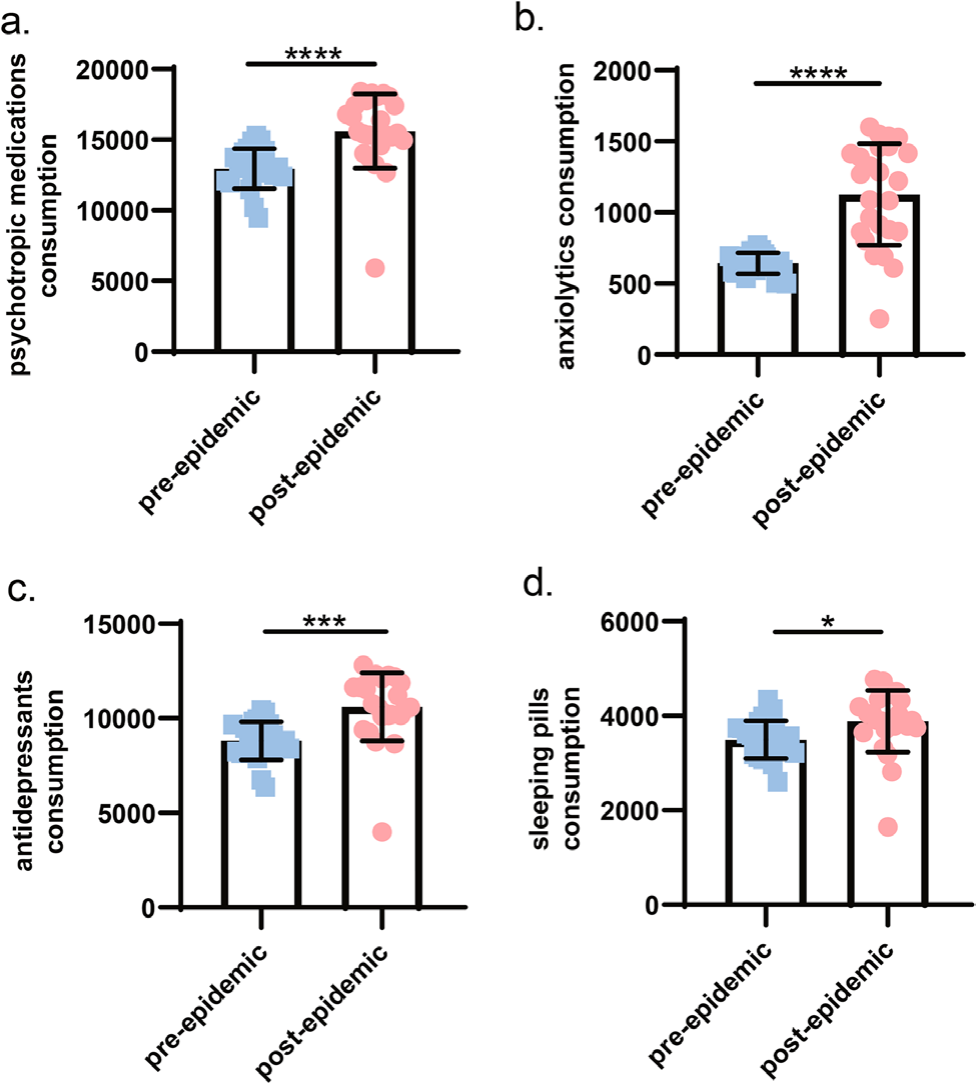
Comparison of drug consumption in the pre-epidemic group and the dur-epidemic group Note: Fig. 4**a**. showed the comparison of psychotropic medications between the the pre-epidemic group and the dur-epidemic group. Anxiolytics mainly include tandospirone and DEA, and antidepressants mainly included SSRI, SNRI and SARI drugs. Figure 4**b**. and Fig. 4**c**. showed the dosage of anxiolytics and antidepressants before and after the epidemic, respectively., the dur-epidemic group was higher than the pre-epidemic group (P < = 0.0001). Sleeping pills collected mirtazapine, benzodiazepines and non-benzodiazepines. Similarly, the consumption of sleeping pills in the dur-epidemic group was higher than that in the pre-epidemic group (Fig. 4**d**., P = 0.0165)

In addition, drug consumption was also an indirect indicator of disease visits. Therefore, we collected the relevant drug consumption data for three types of mental and psychological diseases from 2018 to 2021. These psychotropic medications included anxiolytics, antidepressants, and sleeping pills. In general, the consumption of psychotropic drugs after the COVID-19 outbreak was significantly greater than that before the outbreak, and there was a statistical difference (Fig. 4a., P < 0.0001). Figure 4b-d. showed the differences between the pre-epidemic group and the dur-epidemic group for anxiolytics, antidepressants, and sleeping pills, respectively.

**Fig. 5:**
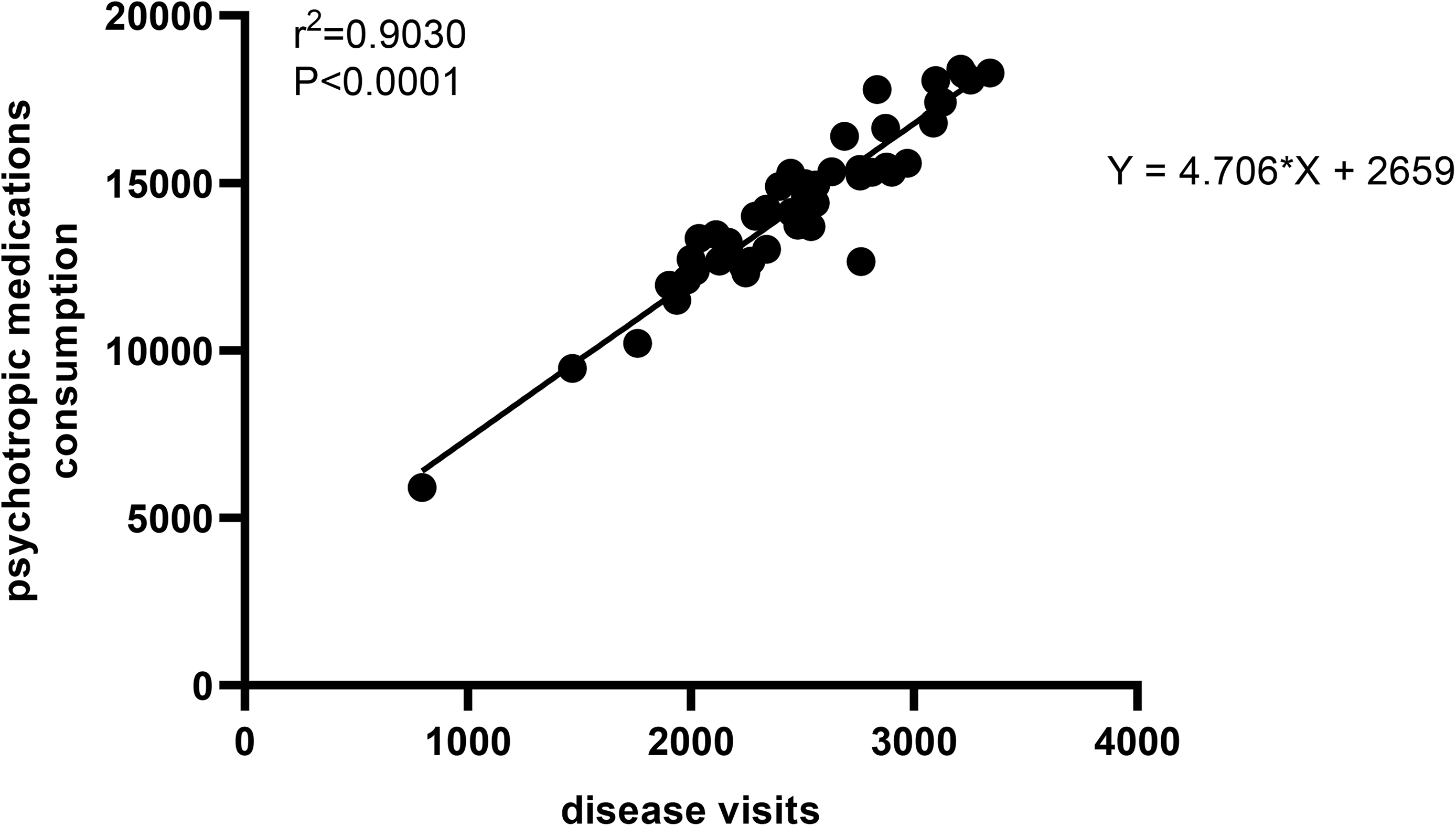
Linear regression of disease visits and drug consumption

Drugs are related to diseases. Because most of these five types of mental and psychological diseases are chronic diseases, there is a phenomenon that the number of disease visits increases year by year with the increase of time. Therefore, we also analyzed the number of inital diagnosis and the number of disease visits in the above study. However, due to the difficulty of data collection, we only collected the total drug consumption of patients with anxiety, depression, and sleep disorders, and could not accurately collect the drug consumption of patients diagnosed with these three diseases for the first time. Based on this situation, we subdivided the drug consumption into months and conducted a correlation analysis with the number of disease visits. Figure 5 showed that there was a positive correlation between the dosage of drugs and the number of disease visits, and the drugs consumption increased with the increase of the number of disease visits (r^2^ = 0.9030, P < 0.0001). Combined with Figs. 1, 2 and 3, we speculate that after removing the time accumulation factor, the annual new drug consumption after the outbreak will be higher than that before the outbreak. In addition, the high agreement between disease visits and drug consumption confirms the robustness of our data.

**Fig. 6:**
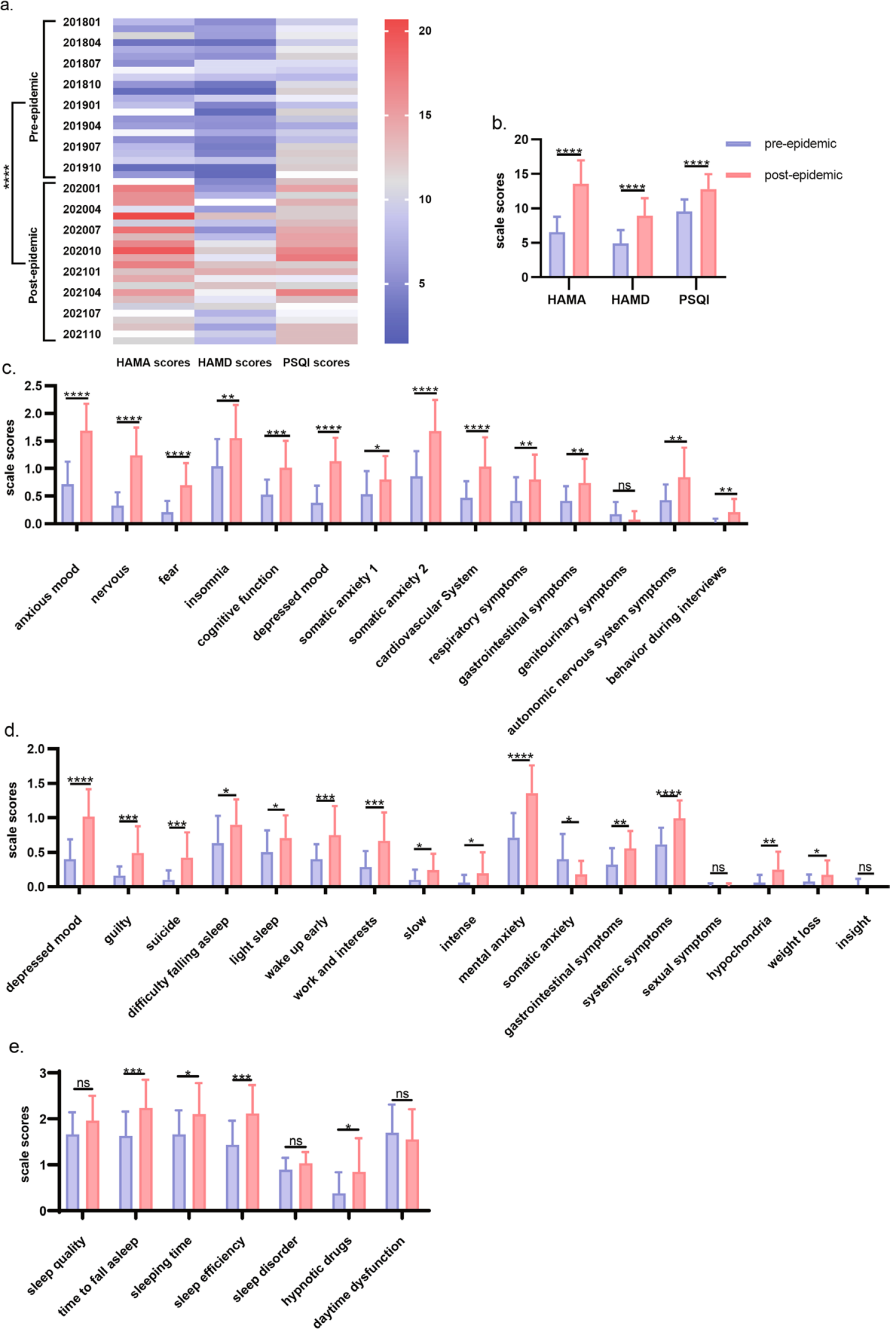
Comparison of scale scores in the pre-epidemic group and the dur-epidemic group Note: Fig. 6**a**. presented the statistical description of HAMA, HAMD and PSQI scores in each month from 2018 to 2021. Figure 6**b**. showed the comparison of HAMA, HAMD and PSQI scores between the pre-epidemic group and the dur-epidemic group. Figure 6**c-e**. showed the comparison of each subitem of HAMA, HAMD and PSQI between the pre-epidemic group and the dur-epidemic group

Scale scores can also reflect disease status. Figure 6a. provided a statistical description of HAMA, HAMD, and PSQI scales scores. Taking December 2019 as the cut-off point, the scale data were divided into the pre-epidemic group and the dur-epidemic group. We compared the total scores of HAMA, HAMD and PSQI between the two groups and found that the total scores of the three scales in the dur-epidemic group were higher than those in the pre-epidemic group, and there were significant difference (Fig. 6a and b., P < 0.0001). We also compared the sub-items (n = 38) of the three scales and found that most (n = 32) were significantly different (P < 0.05), and only 1 subitem in HAMA, 2 subitems in HAMD and 3 subitems in PSQI showed no significant difference (Fig. 6c.-6e.).

Finally, we stratified patients with five types of mental and psychological diseases by gender and age, and explored how patients of different genders and ages are affected by the epidemic. Supplementary Table 2 showed the composition ratios of different genders and ages of patients with five types of mental and psychological diseases in pre-epidemic group and dur-epidemic group. We found that these five types of mental and psychological diseases were all prevalent in women, and the diseases that occur in different age groups were different.

## Discussion

The increasing trend in initial diagnoses, disease visits and growth rates for the five types of mental and psychological disorders suggested that the impact of COVID-19 was long-term. It prompted us to remain vigilant about people’s mental health in this moment.

The world is going through a pandemic caused by the COVID-19, which caused by the novel coronavirus SARS-CoV-2. The severity of the pandemic and its negative messages can lead to people’s insecurities and fears, which are exacerbated by the lack of effective control measures and treatment mechanisms [15–17]. Many countries have imposed lockdowns during the pandemic to prevent widespread infection. These recommendations and enforcement actions are necessary to prevent the spread of the virus, but at the same time they may increase the psychological and mental burden on people [18]. There have been many studies on the COVID-19 pandemic and mental health, but the results were inconsistent, which because these results were limited by the research area, research time and research method.

### COVID-19 pandemic and anxiety and depressive disorder

Anxiety and depressive disorders are common psychiatric problems and occur across all age groups. During the COVID-19 pandemic, there has been a growing awareness of the emotional impact of the pandemic. Researchers in China have investigated the general population’s psychological and mental responses during the COVID-19 pandemic. And they found that more than half of respondents rated the psychological and mental impact of the COVID-19 pandemic as moderate or severe, 16.5% reported moderate to severe depressive symptoms, and about a third reported moderate to severe anxiety symptoms [19]. Another research team in China sampled and analyzed the online posts of about 18,000 Chinese social media users before and after the COVID-19 outbreak. The researchers found that negative emotions such as anxiety, depression, and anger increased, while positive emotions and life satisfaction decreased [20]. Besides, Japan also reported 15 similar data, and its economy took a huge hit [21]. Studies have also reported on the psychological and mental profile of patients with (or suspected) COVID-19 infection and found that they may experience strong emotional and behavioral responses, such as fear, boredom, loneliness, anxiety, insomnia, or anger, which may evolve depression, anxiety, post-traumatic stress, or paranoia, and may even lead to suicide [22, 23]. And these conditions were especially prevalent among patients who were quarantined because their psychological and mental distress tend to be higher [24]. Our study found an upward trend in both annual disease inital diagnosis and disease growth rates for anxiety and depressive disorders, with peaks in 2021. But in terms of the number of visits for anxiety, we found a downward trend in 2020, which may be related to the shock of the outbreak of the COVID-19.

### COVID-19 pandemic and sleep disorders

COVID-19-related illnesses can cause insomnia in patients, which can alter the immune system and negatively impact health. Thus, this prompted us to focus on the relationship between the COVID-19 pandemic and sleep disorders. Exposure to stress, such as social limitations and changes in daily life, accompanied by various sleep disturbances, is known as the phenomenon of coronary insomnia. For healthy people in quarantine, lifestyle changes, fear of infection, and reduced ability to cope with stress contribute to insomnia [25]. Psychosocial and mental stress can also affect sleep patterns and lead to a worsening of individual sleep quality [26. A paper reported that the COVID-19 pandemic will affect circadian rhythms, leading to sleep-wake disturbances [27]. Among individuals who self-isolated during the COVID-19 pandemic, a research team found that isolated individuals generally had higher anxiety and stress levels and lower sleep quality [19]. In addition to implementing epidemiological and preventive measures, sleep hygiene should be promoted as a comprehensive response strategy to the COVID-19 pandemic. Combined with existing research, we concluded that the COVID-19 pandemic harms sleep. In this regard, we also conducted surveys and confirmed that sleep disorders growth rates, drug consumption, and scale scores all increased significantly after the outbreak.

### COVID-19 pandemic and stress disorders

The stressors associated with the COVID-19 pandemic is numerous, including fear of getting sick and dying, fear of infecting others, grief related to the loss of a loved one, social isolation from family and friends, and loss of jobs, business, and income [28]. People living in stressful environments for a long time are more likely susceptible to mental illness. Data from various sources suggested that the current impact of the COVID-19 pandemic can manifest itself as an increase in psychiatric symptoms, such as pandemic-related trauma and stressor-related disorder (TSRD) symptoms, as well as an increase in suicidal ideation and drug consumption [29, 30]. This study also studied the COVID-19 pandemic and stress disorder and found that the number of the inital diagnosis, the number of disease visits, and disease growth rates for stress disorders after the outbreak increased.

### COVID-19 pandemic and eating-excretion disorders

The COVID-19 pandemic has had a profound impact on the severity of symptoms in eating-excretion Disorders, with lack of exercise, reduced income, increased stress, and lack of in-person health care all appearing to have contributed to this development. The relationship between the COVID-19 pandemic and eating-excretion disorders is one of the current hotspots. Many studies have been conducted, but the results were inconsistent. A study has reported a sharp rise in hospital admissions for eating-excretion disorders across Europe during the CQVID-19 pandemic [12]. Other studies have also been reported elevated levels of abnormal disorders-related behaviors in patients with eating disorders, showing marked worsening of symptoms [31–33]. However, there was also evidence that some people with eating disorders didn’t show significant worsening of symptoms due to lack of work and social stress during isolation [31]. Another study suggested that psychological and mental stress due to forced isolation affected the course of gastrointestinal symptoms in patients with functional gastrointestinal diseases, and the findings found that most of the patients’ upper gastrointestinal symptoms improved during the COVID-19 isolation period [13]. Consistent with this, the second wave of studies identified a protective effect of the COVID-19 pandemic on Eating-Excretion Disorders [34]. Therefore, whether the impact of the COVID-19 pandemic on eating-excretion disorders is good or bad prompted us to study it. We found no significant changes in the number of annual inital diagnoses and the number of growth rates of eating-excretion disorders over these 4 years. We also compared the number of the inital diagnosis for eating-excretion disorders between the two groups of the pre-epidemic group and the dur-epidemic group and found no significant difference.

### Medications and scales

Drug consumption and scale scores can reflect the speed of disease growth from different aspects, and they are more concise and objective. Therefore, we investigated drug consumption and scale scores for three common mental and psychological diseases: anxiety, depression, and sleep disorders. We found that after the COVID-19 outbreak, the consumption of anxiolytics, antidepressants, and sleeping pills, as well as the scores of HAMA, HAMD, and PSQI all increased, which once again illustrated the growth trend of related diseases. And the high correlation between drug consumption and the number of disease visits also validated the robustness of the data.

### Gender and age

Previously, scholars reviewed research in the field of gender and mental health and found that women were disproportionately affected by common mental disorders and comorbid mental disorders [35]. A British study found that women are at a disadvantage in mental and psychological diseases, as women are more susceptible to them, and more likely to lead to crime [36]. Another study showed that the lifetime prevalence of mental and psychological diseases was higher in women and that age-sex patterns of mental and psychological diseases were observed in women before menopause [37]. In addition, the relationship between age and mental and psychological diseases has long been reported. The incidence of different mental and psychological diseases is inconsistent in different age groups. For anxiety, the incidence was higher in women than in men between adulthood and menopause, but there was no difference in the other two time periods [37]. moreover, there is a well-known age-sex difference in depression prevalence. Depression became more common in women than men after age 13 (but gender did not affect it before age 13) [37]. Sleep disorders are also linked to age, one study compared specific sleep disorders in 97 patients aged 61–81 years with specific sleep disorders in 264 middle-aged (41–60 years) and 202 young (20–40 years) patients. It was found that most young and middle-aged patients complained of excessive daytime sleepiness, and older adults complained of insomnia as frequently as excessive daytime sleepiness. One assessment showed that 93% of older patients had objective findings, but only 77% of younger patients had objective findings [38]. The prevalence of nearly all mental and psychological diseases increases with age, while the prevalence of post-traumatic stress disorder (PTSD) decreases with age [39]. The above four mental diseases are more common in middle-aged and elderly people, while eating-excretion disorders are more of a disease that young people suffer from. In summary, it was found that the incidence of these five types of mental and psychological diseases were strongly affected by gender and age, and these prevalence were not the same. Therefore, this study also investigated this, reporting the performance of mental and psychological diseases in different gender and age groups before and after the COVID-19 outbreak.

### Strengths and limitations

Strengths: First, the area where the research unit is located was the second most affected area in the country at the beginning of the COVID-19 outbreak, so it has the characteristics of high representativeness. Second, the research unit is the largest public medical institution in the region, so it has the characteristics of a wide range of radiation. Third, the sample size of this study is large, so the research results are more reliable. Finally, the data has a large time span, so it can illustrate the long-term impact of the COVID-19.

Limitation: This study is only a single-center study, so it cannot reflect the situation in the whole country or even the whole world. More research is still needed to focus on this.

## Conclusion

During epidemics, mental health is often affected in more people than infected. Past tragedies, as well as our research, suggest that the effects of pandemics on mental and psychological health may last longer and be more prevalent than the pandemic itself. Therefore, we can expect that after the pandemic subsides, there is a high probability that mental and psychological diseases related to the COVID-19 pandemic, such as anxiety, depression, sleep disorders, PTSD, and drug consumption will increase significantly [28]. Thus, whether or not the pandemic is and is fading, at this moment, our attention to mental and psychological diseases cannot be reduced.

### Electronic supplementary material

Below is the link to the electronic supplementary material.

**Supplementary file 1. Supplementary Table 1**. Statistical description of the disease initial diagnosis and disease visits between 2018-2021. **Supplementary Table 2**a. Comparison of the initial diagnosis of anxiety patients of different ages and genders between pre-epidemic group and dur-epidemic group. **Supplementary Table 2**b. Comparison of the initial diagnosis of depression patients of different ages and genders between pre-epidemic group and dur-epidemic group. **Supplementary Table 2**c. Comparison of the initial diagnosis of sleep disorders patients of different ages and genders between pre-epidemic group and dur-epidemic group. **Supplementary Table 2**d. Comparison of the initial diagnosis of stress disorders patients of different ages and genders between pre-epidemic group and dur-epidemic group. **Supplementary Table 2**e. Comparison of the initial diagnosis of eating and excretion disorders patients of different ages and genders between pre-epidemic group and dur-epidemic group.

## Data Availability

Access to the original data of this study was subject to permission from the First Affiliated Hospital of Wenzhou Medical University.
