# Supplementary material for: Long-term effects of the COVID-19 pandemic on five mental and psychological disorders: in terms of the number of disease visits, drug consumption, and scale scores
Source: BMC Psychiatry. 2023 Sep 18;23:677. doi: 10.1186/s12888-023-05166-0 (PMC10506188; doi:10.1186/s12888-023-05166-0)
Supplement: Supplementary file 1 — Supplementary Table 1. Statistical description of the disease initial diagnosis and disease visits between 2018-2021. Supplementary Table 2a. Comparison of the initial diagnosis of anxiety patients of different ages and genders between pre-epidemic group and dur-epidemic group. Supplementary Table 2b. Comparison of the initial diagnosis of depression patients of different ages and genders between pre-epidemic group and dur-epidemic group. Supplementary Table 2c. Comparison of the initial diagnosis of sleep disorders patients of different ages and genders between pre-epidemic group and dur-epidemic group. Supplementary Table 2d. Comparison of the initial diagnosis of stress disorders patients of different ages and genders between pre-epidemic group and dur-epidemic group. Supplementary Table 2e. Comparison of the initial diagnosis of eating and excretion disorders patients of different ages and genders between pre-epidemic group and dur-epidemic group. [file 12888_2023_5166_MOESM1_ESM.docx]

Supplementary Table 1. Statistical description of the disease initial diagnosis and disease visits between 2018-2021.

| Variables | | 2018 | 2019 | 2020 | 2021 |
| --- | --- | --- | --- | --- | --- |
| Disease first diagnosis, n |  |  |  |  |  |
|  | Anxiety | 2317 | 2442 | 2442 | 2643 |
|  | Depression | 2562 | 3315 | 3426 | 4289 |
|  | Sleep Disorders | 3519 | 3469 | 4913 | 4396 |
|  | Stress Disorders | 274 | 268 | 319 | 383 |
|  | Eating-Excretion Disorders | 79 | 79 | 73 | 77 |
| Disease visits, n |  |  |  |  |  |
|  | Anxiety | 7413 | 8698 | 8562 | 9503 |
|  | Depression | 7740 | 10423 | 11028 | 13711 |
|  | Sleep Disorders | 8995 | 10131 | 10425 | 12473 |
|  | Stress Disorders | 520 | 551 | 688 | 858 |
|  | Eating-Excretion Disorders | 183 | 225 | 209 | 187 |
|  | all mental and psychological diseases | 250154 | 259585 | 240520 | 264670 |

Supplementary Table 2a. Comparison of the initial diagnosis of anxiety patients of different ages and genders between pre-epidemic group and dur-epidemic group.

| Anxiety | Pre-epidemic | Post-epidemic | P value |
| --- | --- | --- | --- |
| Gender, male, n (%) | 1527 (33.46) | 1805 (34.18) | 0.455 |
| Age, n (%) |  |  | <0.0001 |
| <18 | 227 (3.01) | 477 (9.03) |  |
| 18-44 | 1698 (34.68) | 1737 (32.89) |  |
| 45-64 | 1983 (45.39) | 2178 (41.24) |  |
| >=65 | 655 (16.92) | 889 (16.83) |  |

Notes: Frequencies (percentages) were used to describe categorical variables. Categorical variables were compared using the χ2 test and the Wilcoxon test. Supplementary Table 2a. showed that although there were statistical differences in age groups between two groups, the patients diagnosed with anxiety were mainly middle-aged women.

Supplementary Table 2b. Comparison of the initial diagnosis of depression patients of different ages and genders between pre-epidemic group and dur-epidemic group.

| Depression | Pre-epidemic | Post-epidemic | P value |
| --- | --- | --- | --- |
| Gender, male, n (%) | 1952 (35.20) | 2657 (33.02) | 0.008 |
| Age, n (%) |  |  | <0.0001 |
| <18 | 1124 (20.27) | 2334 (29.00) |  |
| 18-44 | 2664 (48.05) | 3594 (44.66) |  |
| 45-64 | 1290 (23.26) | 1480 (18.40) |  |
| >=65 | 467 (8.42) | 639 (7.94) |  |

Notes: Frequencies (percentages) were used to describe categorical variables. Categorical variables were compared using the χ2 test and the Wilcoxon test. Supplementary Table 2b. showed that the epidemic affected the gender and age distribution of depression patients (P<0.05), which showed that the proportion of women and minors increased after the outbreak.

Supplementary Table 2c. Comparison of the initial diagnosis of sleep disorders patients of different ages and genders between pre-epidemic group and dur-epidemic group.

| Sleep disorders | Pre-epidemic | Post-epidemic | P value |
| --- | --- | --- | --- |
| Gender, male, n (%) | 2369 (36.19) | 3430 (35.71) | 0.533 |
| Age, n (%) |  |  | <0.0001 |
| <18 | 154 (2.30) | 396 (4.12) |  |
| 18-44 | 2156 (32.22) | 3540 (36.85) |  |
| 45-64 | 3169 (47.36) | 4355 (45.34) |  |
| >=65 | 1212 (18.12) | 1315 (13.69) |  |

Notes: Frequencies (percentages) were used to describe categorical variables. Categorical variables were compared using the χ2 test and the Wilcoxon test. Supplementary Table 2c. showed that although there were statistical differences in age groups between two groups, the patients diagnosed with sleep disorders were mainly middle-aged women.

Supplementary Table 2d. Comparison of the initial diagnosis of stress disorders patients of different ages and genders between pre-epidemic group and dur-epidemic group.

| Stress disorders | Pre-epidemic | Post-epidemic | P value |
| --- | --- | --- | --- |
| Gender, male, n (%) | 217 (42.47) | 269 (36.70) | 0.040 |
| Age, n (%) |  |  | 0.163 |
| <18 | 270 (52.84) | 434 (59.21) |  |
| 18-44 | 146 (28.57) | 185 (25.24) |  |
| 45-64 | 73 (14.29) | 89 (12.14) |  |
| >=65 | 22 (4.30) | 25 (3.41) |  |

Notes: Frequencies (percentages) were used to describe categorical variables. Categorical variables were compared using the χ2 test and the Wilcoxon test. Supplementary Table 2d. indicated that the patients diagnosed with stress disorders after the epidemic were mainly female (P<0.05).

Supplementary Table 2e. Comparison of the initial diagnosis of eating and excretion disorders patients of different ages and genders between pre-epidemic group and dur-epidemic group.

| Eating-Excretion Disorders | Pre-epidemic | Post-epidemic | P value |
| --- | --- | --- | --- |
| Gender, male, n (%) | 23 (15.33) | 18 (11.39) | 0.309 |
| Age, n (%) |  |  | 0.309 |
| <18 | 37 (24.67) | 41 (25.94) |  |
| 18-44 | 100 (66.67) | 105 (66.46) |  |
| 45-64 | 11 (7.33) | 6 (3.80) |  |
| >=65 | 2 (1.33) | 6 (3.80) |  |

Notes: Frequencies (percentages) were used to describe categorical variables. Categorical variables were compared using the χ2 test and the Wilcoxon test. Supplementary Table 2e. indicated that young females were the main ones with eating and excretion disorders, and there was no statistical difference between pre-epidemic group and dur-epidemic group (P>0.05).
